# Radar Target Tracking for Unmanned Surface Vehicle Based on Square Root Sage–Husa Adaptive Robust Kalman Filter

**DOI:** 10.3390/s22082924

**Published:** 2022-04-11

**Authors:** Shuanghu Qiao, Yunsheng Fan, Guofeng Wang, Dongdong Mu, Zhiping He

**Affiliations:** 1 College of Marine Electrical Engineering, Dalian Maritime University, Dalian 116026, China; qiaosh197865@163.com (S.Q.); yunsheng@dlmu.edu.cn (Y.F.); ddmu@dlmu.edu.cn (D.M.); zhipinghe@dlmu.edu.cn (Z.H.); 2 Key Laboratory of Technology and System for Intelligent Ships of Liaoning Province, Dalian 116026, China

**Keywords:** target tracking, unmanned surface vehicle, Sage–Husa adaptive Kalman filter, square root Sage–Husa adaptive robust Kalman filter, position, velocity

## Abstract

Dynamic information such as the position and velocity of the target detected by marine radar is frequently susceptible to external measurement white noise generated by the oscillations of an unmanned surface vehicle (USV) and target. Although the Sage–Husa adaptive Kalman filter (SHAKF) has been applied to the target tracking field, the precision and stability of SHAKF remain to be improved. In this paper, a square root Sage–Husa adaptive robust Kalman filter (SR-SHARKF) algorithm together with the constant jerk model is proposed, which can not only solve the problem of filtering divergence triggered by numerical rounding errors, inaccurate system mathematics, and noise statistical models, but also improve the filtering accuracy. First, a novel square root decomposition method is proposed in the SR-SHARKF algorithm for decomposing the covariance matrix of SHAKF to assure its non-negative definiteness. After that, a three-segment approach is adopted to balance the observed and predicted states by evaluating the adaptive scale factor. Finally, the unbiased and the biased noise estimators are integrated while the interval scope of the measurement noise is constrained to jointly evaluate the measurement and observation noise for better adaptability and reliability. Simulation and experimental results demonstrate the effectiveness of the proposed algorithm in eliminating white noise triggered by the USV and target oscillations.

## 1. Introduction

Marine radar is widely exploited as a navigational sensing device that can gather dynamic information on surrounding vessels over a wide area while maintaining a decent detection performance [1]. However, its underlying technology is sensitive to external disturbances like ocean waves, which will lead to inaccurate dynamic information being obtained [2,3,4]. To address this issue, target tracking technology has been successfully implemented to predict the dynamic information of the target and produce generally accurate output results. Target tracking is the forecast of the ship’s next possible trajectory as a function of its previous coordinates. The accuracy of the prediction is determined by the accuracy with which the target’s prior and present positions were measured [5]. It is easy to realize the compensation of measurement errors and this plays a vital role in collision avoidance and search and rescue [6].

The Kalman filter (KF) algorithm [7,8] is capable of estimating certain conditions of dynamic systems either in the past, present, or future, or unknown characteristics of the model, and has been widely used in target tracking. Zhang et al. [9] proposed a modified KF in which a novel fading factor is introduced into the cubature KF method to achieve higher robustness and reduce the algorithm complexity simultaneously. The KF is optimized using a new optimization criterion that is integrated with the least-squares method for bearings-only maneuvering target tracking [10]. Lu et al. [11] combined the KF and variational Bayesian method to derive a new robust KF that is more resilient and adaptable than the existing related filters. To tackle the instability problem caused by large-scale application variations, Feng et al. [12] presented a novel filter structure that integrates KF with spatial-temporal regularized correlation filters. To cope with the problem of heavy noise causing dramatic performance degradation, Liu et al. [13] proposed two new maximum correntropy extended KF algorithms that apply the maximum correntropy criterion rather than the minimal mean-square-error criterion to the extended KF. For the preceding target vehicle lateral state estimation, Zhou et al. [14] integrated sensor data and estimated states as measurements and developed a state-input-parameter mixed KF to deal with the cohabitation of uncertain model parameters and unknown control inputs.

The performance of a traditional KF may suffer due to the undervaluation of the noise covariance matrix and the response latency of the gain matrix when the target undergoes an abrupt state change. To overcome the aforementioned problems, SHAKF [15,16] has been introduced. To fulfill the need for greater accuracy and stability of the integrated navigation system, Guo et al. [17] implemented a Sage–Husa adaptive Kalman filter with nonholonomic constraints and forward/backward filtering to an IMU/GPS integrated system. In [18], a novel approach based on the adaptive current statistical model with velocity prediction is proposed, which uses a simplified Sage–Husa estimator to achieve an online measurement noise covariance matrix. At each tracking step, Hou et al. [19] employed a modified Sage–Husa online noise estimator to predict the uncertain process and measurement noise. To improve the performance of state estimation, a state error covariance adaptive five-degree cubature Kalman technique based on the Sage–Husa noise estimation principle is presented in [20]. Yan et al. [21] proposed a tracking method that integrates an improved compressive tracking algorithm with a modified SHAKF to achieve excellent accuracy and robustness in tracking. Luo et al. [22] proposed an adaptive unscented KF based on a modified SHAKF and divergence calculation technique to correct the measurement noise for multi-dimensional vehicle driving state evaluation. To improve the accuracy of an attitude heading reference system, Narasimhappa et al. [23] proposed a modified Sage–Husa adaptive robust KF (MSHARKF) based on adaptive robust estimate theory. However, due to the small size of a USV and its susceptibility to external environmental factors, these methods are not very effective in eliminating white noise generated by the oscillations of the USV and the target, and this will give rise to instability and loss of accuracy when dealing with the filter divergence problem that occurs in target tracking. Therefore, the square root Sage–Husa adaptive robust KF (SR-SHARKF) algorithm is used for target tracking of the constant jerk model in this paper.

In this paper, the contributions are primarily reflected in five aspects as follows:

(1) Use of the constant jerk model can compensate for the dynamic interference caused by a large number of external factors in the marine environment.

(2) A SR-SHARKF algorithm is developed to significantly improve the filtering accuracy while reducing the impact of transient disturbance caused by the large-scale USV jitter problem on subsequent estimation.

(3) A novel square-root decomposition method is introduced into the SHAKF algorithm to decompose the covariance matrix to assure its non-negative definiteness, which can address the issue of filtering divergence triggered by numerical rounding errors.

(4) A three-segment approach is adopted to balance the observations and predicted states by evaluating the adaptive scale factor, which is beneficial to reduce the influence of outliers and state disturbances generated by severe conditions of the USV oscillations.

(5) The measurement noise range is limited to a minimum and maximum threshold, after which the unbiased and biased noise estimators are integrated to evaluate the measurement and observation noise, resulting in increased flexibility and reliability by minimizing filtering divergence.

The remainder of the paper is laid out as follows: Section 2 briefly introduces the state-space model of the target tracking system. Section 3 describes briefly the Sage–Husa adaptive Kalman filter algorithm and the extended Sage–Husa adaptive Kalman filter is proposed. In Section 4, the parameters are determined and we compare the results of simulations with different algorithms. Finally, the conclusions are presented in Section 5.

## 2. State-Space Model of Target Tracking System

The acceleration of the target may be constantly changing during the movement, but it can be considered that the change rate of acceleration, i.e., the jerk, remains a constant value over a long period and space, and subject to random systematic errors within a certain range. Therefore, the SR-SHARKF algorithm is applied based on the constant jerk dynamic model. Since the algorithm is proposed for linear systems, it is only applicable to linear systems. The constant jerk dynamic model comprises all the indispensable parameters for target tracking, and it is used to link the radar-measured variables with the state variables.

**Figure 1:**
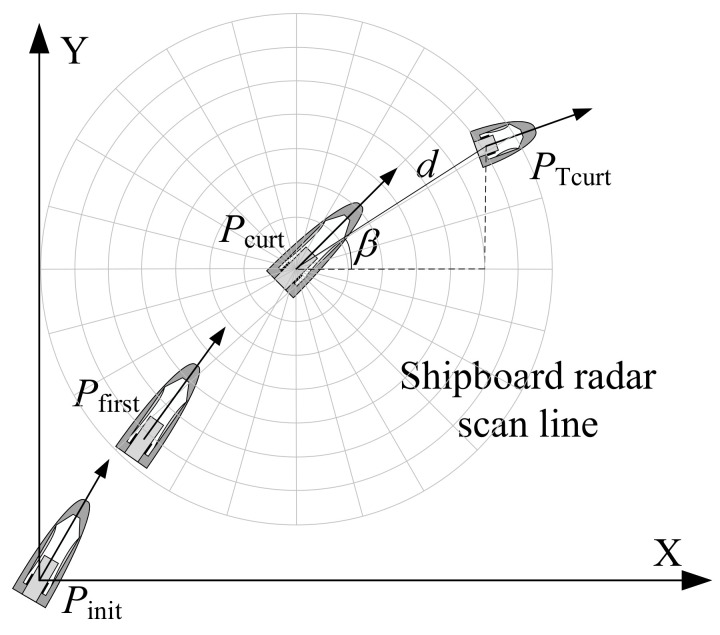
Coordinate systems for target parameterization with radar sensor measurements.

Traditional KF-based tracking algorithms are typically accomplished by tracking the target’s coordinates, whether applied to shipboard radar systems or other platforms. Since the information measured by the radar includes only the velocity of the target, the range from the USV to the target, the azimuth, and the heading of the target, the aforementioned parameters should be converted to Cartesian coordinates to apply the KF framework. The mapping of angles and ranges to Cartesian space has an uncertainty factor that causes many errors, but they are within the allowable range of the system. Assuming that the initial starting point $P_{\mathrm{init}}$ of the USV radar target tracking is the coordinate origin, the orientation of due east is the X-axis, and the orientation of due north is the Y-axis, a 2-D plane coordinate system in the radar plane is established as shown in Figure 1. The coordinates of the USV’s initial starting point $P_{\mathrm{init}}$, and the first point $P_{\mathrm{first}}$ to the current point $P_{\mathrm{curt}}$ are $\left(x_{0}^{U},y_{0}^{U}\right)$, $\left(x_{1}^{U},y_{1}^{U}\right)$,…,$\left(x_{n}^{U},y_{n}^{U}\right)$, respectively. Considering that $P_{\mathrm{Tcurt}}\left(x,y\right)$ represents the current position of the marine target, the current position of the target can be obtained from the position of the USV as follows:

(1) $$\left\{\begin{array}{c} x=\sum_{i=1}^{n}\left(x_{i}^{U}-x_{i-1}^{U}\right)+d\cos\beta \\ y=\sum_{i=1}^{n}\left(y_{i}^{U}-y_{i-1}^{U}\right)+d\sin\beta \end{array}\right.$$

where *n* represents the number of the radar’s detection periods from the start to the current state for target tracking, *d* is the distance between the USV and the target, and β is expressed as

(2) $$\beta \;=\;\left\{\begin{array}{c} \frac{5\pi }{2}-\theta ,\;\;\;if\;\;\left(\frac{5\pi }{2}-\theta \right)\leq 2\pi \;\\ \frac{\pi }{2}-\theta ,\;\;\;\;\;\;elsewise \end{array}\right.$$

where θ is the azimuth between the target and the USV.

Let the relative position, relative velocity, relative acceleration, and relative jerk in the X-axis direction be $x_{k}$, ${\dot{x}}_{k}$, ${\ddot{x}}_{k}$, ${\dddot{x}}_{k}$, and the measured values of the relative position and relative velocity are $x_{km}$, ${\dot{x}}_{km}$, respectively. The system state-space model of the target can be established as the following discrete formation. The system equation is

(3) $$X_{k}\;=\;\Phi X_{k-1}+GW_{k-1}$$

where $X_{k}$ is the estimated state vector of the target motion in front of the *k*-th detection period, Φ is the state transition matrix, *G* represents the system noise distribution matrix, and $W_{k}$ denotes the system noise vector. The matrix forms of $X_{k}$, Φ, and *G* are expressed as follows:

(4) $$X_{k}={\left[x_{k},{\dot{x}}_{k},{\ddot{x}}_{k},{\dddot{x}}_{k}\right]}^{T}$$

(5) $$\Phi \;=\;\left[\begin{array}{cccc} 1 & \Delta T & \Delta T^{2}/2 & \Delta T^{3}/6\\ 0 & 1 & \Delta T & \Delta T^{2}/2\\ 0 & 0 & 1 & \Delta T\\ 0 & 0 & 0 & 1 \end{array}\right]$$

(6) $$G\;=\;{\left[\begin{array}{cccc} \Delta T^{4}/24 & \Delta T^{3}/6 & \Delta T^{2}/2 & \Delta T \end{array}\right]}^{T}$$

where ΔT is the radar sampling time. The measurement equation is

(7) $$Z_{k}=HX_{k}+V_{k}$$

where $Z_{k}$ is the measurement state vector of the target motion in front of the *k*-th detection period, *H* is the measurement matrix, and $V_{k}$ represents the measurement noise vector. The matrix forms of $Z_{k}$ and *H* are denoted as follows:

(8) $$Z_{k}={\left[z_{k},{\dot{z}}_{k}\right]}^{T}$$

(9) $$H\;=\;\left[\begin{array}{cccc} 1 & 0 & 0 & 0\\ 0 & 1 & 0 & 0 \end{array}\right]$$

In (3) and (7), both $W_{k}$ and $V_{k}$ denote Gaussian white noise that satisfies $W_{k}∼WN\left(q_{k},Q_{k}\right)$, $V_{k}∼WN\left(r_{k},R_{k}\right)$; they are independent of each other and meet the following requirements:

(10) $$E\left[W_{k}\right]=q_{k}$$

(11) $$Cov\left[W_{k},{W_{j}}^{T}\right]=\left\{\begin{array}{c} Q_{k},j=k\\ 0,\;\;\;\;\;\;\;\;\;\;j\neq k \end{array}\right.$$

(12) $$E\left[V_{k}\right]=r_{k}$$

(13) $$Cov\left[V_{k},{V_{j}}^{T}\right]=\left\{\begin{array}{c} R_{k},j=k\\ 0,\;\;\;\;\;\;\;\;\;\;j\neq k \end{array}\right.$$

(14) $$Cov\left[W_{k},{V_{j}}^{T}\right]=0$$

where $q_{k}$ and $Q_{k}$ represent the mean and covariance of the system noise, respectively, and $r_{k}$, $R_{k}$ denote the mean and covariance of the measurement noise.

The model in the Y-axis direction can be obtained in the same way, so this paper will not describe it in detail here.

## 3. Proposed Square-Root Sage–Husa Adaptive Robust Kalman Filter (SR-SHARKF)

### 3.1. The SHAKF Algorithm

SHAKF, one of variants of the conventional KF, has a comparative advantage in eliminating noise that can vary with environmental conditions, which is significant in increasing the target tracking system’s stability and accuracy. The filtering flows of the SHAKF algorithm can be described as follows [24]:

(1) Time update

(15) $$X_{k,k-1}=\Phi_{k,k-1}X_{k-1}+G_{k-1}q_{k-1}$$

(16) $$P_{k,k-1}=\Phi_{k,k-1}P_{k-1}\Phi_{k,k-1}^{T}+G_{k-1}Q_{k-1}G_{k-1}^{T}$$

where $P_{k,k-1}$ represents the one-step state prediction error covariance matrix and $P_{k-1}$ denotes the error covariance matrix of state prediction.

(2) Measurement update

(17) $$\varepsilon_{k}=Z_{k}-H_{k}X_{k,k-1}-r_{k-1}$$

(18) $$X_{k}=X_{k,k-1}+K_{k}\varepsilon_{k}$$

(19) $$K_{k}\;=\;P_{k,k-1}H_{k}^{T}{\left(H_{k}P_{k,k-1}H_{k}^{T}+R_{k}\right)}^{-1}$$

(20) $$P_{k}\;=\;\left(I-K_{k}H_{k}\right)P_{k,k-1}$$

where $\varepsilon_{k}$ is the discrepancy vector and $K_{k}$ is the filter gain matrix. The statistical recursive estimator for time-varying noise is

(21) $$G_{k}q_{k}=\left(1-d_{k}\right)G_{k-1}q_{k-1}+d_{k}\left(X_{k}-\Phi_{k,k-1}X_{k-1}\right)$$

(22) $$\begin{array}{c} \begin{array}{cc} G_{k}Q_{k}G_{k}^{T}= & \left(1-d_{k}\right)G_{k-1}Q_{k-1}G_{k-1}^{T}+d_{k}(K_{k}\varepsilon_{k}\varepsilon_{k}^{T}K_{k}^{T}\\ & +P_{k}-\Phi_{k,k-1}P_{k-1}\Phi_{k,k-1}^{T}) \end{array} \end{array}$$

(23) $$r_{k}=\left(1-d_{k}\right)r_{k-1}+d_{k}\left(Z_{k}-H_{k}X_{k,k-1}\right)$$

(24) $$R_{k}=\left(1-d_{k}\right)R_{k-1}+d_{k}\left(\varepsilon_{k}\varepsilon_{k}^{T}-H_{k}P_{k,k-1}H_{k}^{T}\right)$$

where $d_{k}=(1-b)/(1-b^{k})\in \left(0,1\right)$ denotes the amnestic factor, *b* is the forgetting factor, and 0<b<1.

### 3.2. The Square-Root Sage–Husa Adaptive Robust Kalman Filter (SR-SHARKF)

In order to operate on the vibration problem of the USV and target, the SR-SHARKF method is proposed, which is based on the SHAKF algorithm. The improvement of the SR-SHARKF algorithm is mainly split into the following several aspects.

#### 3.2.1. Square-Root Decomposition Method

Based on the SHAKF algorithm, a square-root decomposition method of the state error covariance matrix is proposed. On the SR-SHARKF algorithm, the one-step state prediction error covariance matrix $P_{k,k-1}$, the error variance matrix $P_{k-1}$, and the system noise covariance matrix $Q_{k-1}$ are decomposed. This can guarantee that the covariance matrix is non-negative definite to prevent algorithm divergence issues caused by rounding errors [25].

The covariance decomposition matrix can be expressed as

(25) $$U_{k,k-1}=\left[\Phi_{k,k-1}U_{k-1}\;\;\;\;\;\;\;\;\;\;G_{k-1}S_{k-1}\right]$$

where $U_{k,k-1}$, $U_{k-1}$ and $S_{k-1}$ represent the decomposition factors of matrices $P_{k,k-1}$, $P_{k-1}$ and $Q_{k-1}$ respectively, and satisfy $P_{k,k-1}\;=\;U_{k,k-1}U_{k,k-1}^{T}$, $P_{k-1}\;=\;U_{k-1}U_{k-1}^{T}$, $Q_{k-1}\;=\;S_{k-1}S_{k-1}^{T}$. $U_{k-1}$ and $S_{k-1}$ are obtained by Cholesky factorization [26]:

(26) $$U_{k-1}\;=\;chol\left(P_{k-1}\right)$$

(27) $$S_{k-1}\;=\;chol\left(Q_{k-1}\right)$$

where chol(·) represents the Cholesky decomposition function.

The corresponding measurement update changes as shown

(28) $$F_{k}=U_{k,k-1}^{T}H_{k}^{T}$$

(29) $$\lambda_{k}\;=\;{\left[F_{k}^{T}F_{k}+R_{k}\right]}^{-1}$$

(30) $$K_{k}\;=\;U_{k,k-1}F_{k}\lambda_{k}$$

(31) $$P_{k}\;=\;\left(I-K_{k}H_{k}\right)U_{k,k-1}U_{k,k-1}^{T}$$

After decomposition, although the forms of the covariance matrix, gain matrix, and other matrices have changed, the core idea of the KF is still there, which has a significant effect on overcoming the filter divergence triggered by numerical rounding errors.

#### 3.2.2. Three-Segment Method

The SR-SHARKF algorithm introduces an adaptive scale factor to balance the contribution of kinematic model information and measurements on state vector estimation utilizing a three-segment technique followed by learning statistics [27]. In each iteration, the adaptive scale factor is updated as Kalman gain and state estimation error covariance matrix, reducing the uncertainty in the prediction state error model.

The adaptive scale factor $\alpha_{k}$ is calculated as follows

(32) $$\alpha_{k}\;=\;\left\{\begin{array}{c} 1,\;\;\;\;\;\;\;\;\;\;\;\;\;\;\;\;\;\;\;\;\;\;\;\;\;\;\;\;\;\;\;\;\;\;\;\;\;\;\;\;\;\;\;\;\;\;\;\;\;\;\;\;\;\;\;\;\;\;\;\;\;\;\;\;\;\;\;\;\;\;\;\;\;\;\;\;\;\;\;\;\left|\Delta X_{k}\right|\leq c_{0}\\ \frac{c_{0}}{\;\;\left|\Delta X_{k}\right|}{\left(\frac{c_{1}-\left|\Delta X_{k}\right|}{c_{1}-c_{0}}\right)}^{2},\;\;\;\;c_{0}<\left|\Delta X_{k}\right|\leq c_{1}\;\\ 0,\;\;\;\;\;\;\;\;\;\;\;\;\;\;\;\;\;\;\;\;\;\;\;\;\;\;\;\;\;\;\;\;\;\;\;\;\;\;\;\;\;\;\;\;\;\;\;\;\;\;\;\;\;\;\;\;\;\;\;\;\;\;\;\;\;\;\;\;\;\;\;\;\;\;\;\;\;\;\left|\Delta X_{k}\right|>\;c_{1}\; \end{array}\right.$$

where $\;\Delta X_{k}\;=\;X_{k}-X_{k,k-1}$ denotes the residual vector of the predicted state vector, and $1\leq c_{0}\leq 1.5$ and $3\leq c_{1}\leq 4.5$ are two constants [23,28]. The learning statistics of the predicted state error model $\;\;\left|\Delta X_{k}\right|$ are given as

(33) $$\;\;\left|\Delta X_{k}\right|\;=\;\frac{∥\varepsilon_{k}∥}{\sqrt{\mathrm{tr}(H_{k}P_{k,k-1}H_{k}^{T}+R_{k})}}$$

where tr(·) represents the trace of the matrix. Then, the gain matrix and the error variance matrix are adjusted to

(34) $$\lambda_{k}\;=\;{\left[\frac{1}{\alpha_{k}}F_{k}^{T}F_{k}+R_{k}\right]}^{-1}$$

(35) $$K_{k}\;=\;\frac{1}{\alpha_{k}}U_{k,k-1}F_{k}\lambda_{k}$$

(36) $$P_{k}\;=\;\frac{1}{\alpha_{k}}\left(I-K_{k}H_{k}\right)U_{k,k-1}U_{k,k-1}^{T}$$

#### 3.2.3. Noise Covariance Adjustment Method

According to (17), if the practical system measurement noise is lower than the theoretical one and the preceding estimation error is too great, the *R*-matrix is more likely to lose its positive definiteness, causing the filtering results to diverge. To overcome this problem, the sequential filtering approach is used to limit the size of each element of the *R*-matrix diagonal, and the biased noise estimator is introduced to regulate the *R*-matrix and *Q*-matrix. The biased noise estimator corresponding to the *R*-matrix and *Q*-matrix can be expressed as [29]:

(37) $$R_{k}=\left(1-d_{k}\right)R_{k-1}+d_{k}\left(\varepsilon_{k}\varepsilon_{k}^{T}\right)$$

(38) $$G_{k}Q_{k}G_{k}^{T}=\left(1-d_{k}\right)G_{k-1}Q_{k-1}G_{k-1}^{T}+d_{k}\left(K_{k}\varepsilon_{k}\varepsilon_{k}^{T}K_{k}^{T}\right)$$

The upper and lower bounds of the *R*-matrix named $R_{max}$ and $R_{min}$ are given as follows

(39) $$R_{max}=diag\left(R_{max}^{1},R_{max}^{2},\ldots ,R_{max}^{l}\right)$$

(40) $$R_{min}=diag\left(R_{min}^{1},R_{min}^{2},\ldots ,R_{min}^{l}\right)$$

where *l* is the dimension of measurement vector. The scalar measurement equation is expressed as follows

(41) $$\beta_{k}^{i}=\varepsilon_{k}^{i}{\left(\varepsilon_{k}^{i}\right)}^{T}-H_{k}^{i}P_{k,k-1}^{i}{\left(H_{k}^{i}\right)}^{T}$$

where the superscript *i* denotes that this variable corresponds to the *i*th vector in the *R*-matrix. The *R*-matrix range is limited to meet the following conditions

(42) $$R_{k}^{\left(i\right)}=\left\{\begin{array}{cc} \left(1-d_{k}\right)R_{k-1}^{\left(i\right)}+d_{k}R_{min}^{\left(i\right)}, & \beta_{k}^{i}<R_{min}^{\left(i\right)}\\ R_{max}^{\left(i\right)}, & \beta_{k}^{i}>R_{max}^{\left(i\right)}\\ \left(1-d_{k}\right)R_{k-1}^{\left(i\right)}+d_{k}R_{k,k-1}^{\left(i\right)}, & others \end{array}\right.$$

where $R_{k}^{\left(i\right)}$ represents the *i*th scalar element of the diagonal of the *R*-matrix at time *k*[30]. The unbiased and biased noise estimators are merged, and $R_{k,k-1}^{\left(i\right)}$ can be denoted as follows

(43) $$R_{k,k-1}^{\left(i\right)}\;=\;\left\{\begin{array}{c} \beta_{k}^{i},\;\;\;\;\;\;\;\;\;\;\;\;\;\;\;\;\;\;\;\;\;\;if\;\;R_{k}^{\left(i\right)}\;\;is\;\;positive\;\;definite\;\\ \varepsilon_{k}^{i}{\left(\varepsilon_{k}^{i}\right)}^{T},\;\;if\;\;R_{k}^{\left(i\right)}\;is\;\;not\;\;positive\;\;definite \end{array}\right.$$

The calculation formula of the *Q*-matrix can be expressed as

(44) $$G_{k}Q_{k}G_{k}^{T}=\left\{\begin{array}{c} \mathrm{Equation}\;\left(22\right),\;\;if\;Q_{k}\;\;is\;\;positive\;\;semidefinite\;\;\\ \mathrm{Equation}\;\left(38\right),\;\;if\;\;Q_{k}\;\;is\;\;not\;\;positive\;\;semidefinite \end{array}\right.$$

#### 3.2.4. The SR-SHARKF Algorithm Steps

The SR-SHARKF algorithm validly integrates the square-root decomposition method, three-segment method, and noise covariance adjustment method to obtain a complete set of algorithm update steps, which mainly include the following steps:

Step 1. The initial values $X_{0}$, $P_{0}$, $U_{0}$, $r_{0}$, $R_{0}$, $q_{0}$, $Q_{0}$, $S_{0}$, *b*, $c_{0}$, $c_{1}$, k=1 are given.

Step 2. The nominal state and the error covariance matrix are predicted in one step by (15) and (16) to obtain $X_{k,k-1}$ and $P_{k,k-1}$. $P_{k,k-1}$ is decomposed into $U_{k,k-1}$ using (25).

Step 3. The new information series $d_{k}$, $r_{k}$, $\varepsilon_{k}$ are updated sequentially. If there exists $\beta_{k}^{i}$ that satisfies $\;\beta_{k}^{i}>R_{max}^{\left(i\right)}$ or $\beta_{k}^{i}<R_{min}^{\left(i\right)}$, then the scalar elements in the *R*-matrix according to (42) are updated. If not satisfied, then the elements of the *R*-matrix judge whether the *R*-matrix is positive definite according to (43) to be updated by (42).

Step 4. The adaptive scale factor $\alpha_{k}$ is obtained by (32), and successively uses (34), (28), (35), (18), and (36) to calculate $\lambda_{k}$, $F_{k}$, $K_{k}$, $X_{k}$, and $P_{k}$, respectively.

Step 5. $q_{k}$ and $Q_{k}$ adopting (21) and (44) are updated, and then $P_{k-1}$ and $Q_{k-1}$ are decomposed using (26) and (27) to obtain $U_{k-1}$, $S_{k-1}$.

Step 6. Skip to step 2.

**Figure 2:**
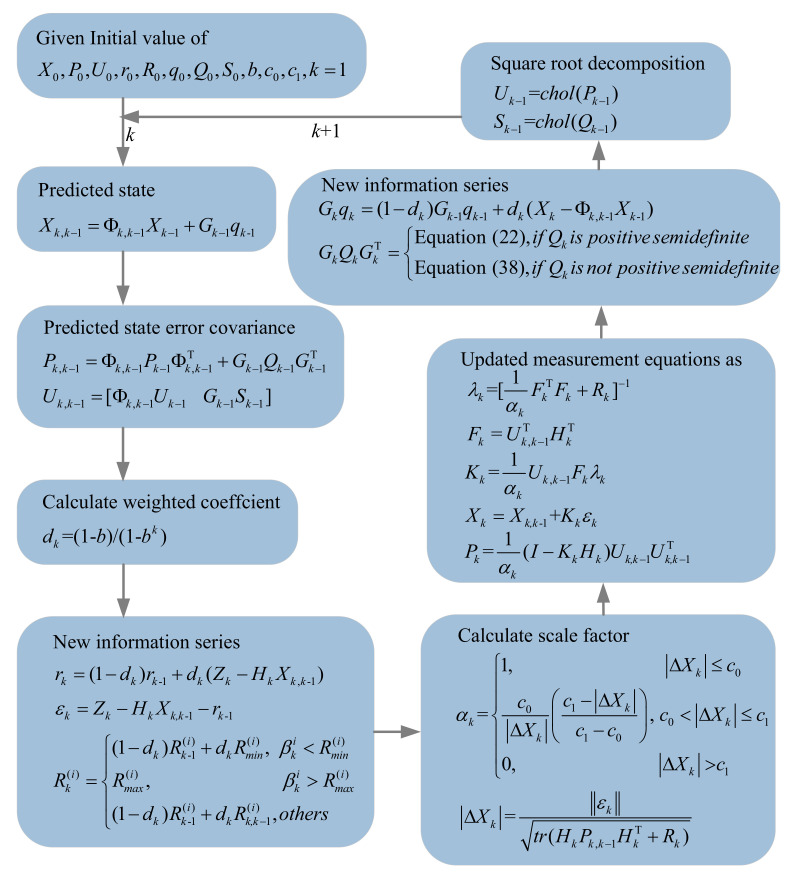
Flow chart of the SR-SHARKF algorithm.

The SR-SHARKF algorithm’s flow chart is presented in Figure 2.

## 4. Simulation and Experimental Results Discussion

Simulations and experiments for the existing standard KF, SHAKF, and MSHARKF [23] with the proposed algorithm were performed to verify and evaluate the effectiveness of the proposed SR-SHARKF algorithm. A simulation test was implemented to compare the performances with the existing algorithms under severe interference from a single-Gaussian distribution and a mixed-Gaussian distribution. Unlike the single-Gaussian distribution, the simulation with mixed Gaussian distribution was operated with 1000 Monte Carlo simulations. Finally, the feasibility of the SR-SHARKF algorithm is discussed by a set of real ship experiment data.

### 4.1. Simulations and Discussions

#### 4.1.1. Simulations and Analysis of the Noise with Single-Gaussian Distribution

In this section, we compare the proposed algorithm with other existing algorithms by using a maneuvering target tracking example that is generated based on the model (3) and (7) with ΔT=2 s and verifies the higher estimate performance of our algorithm. The single-Gaussian process and measurement noise are given as follows:

(45) $$\left\{\begin{array}{c} q_{1k}∼N\left(0,0.005\right)\\ q_{2k}∼N\left(0,0.005\right)\\ q_{3k}∼N\left(0,0.005\right)\\ q_{4k}∼N\left(0,0.005\right)\\ r_{1k}∼N\left(0,1\right)\\ r_{2k}∼N\left(0,1\right) \end{array}\right.$$

In this experiment, the root-mean-square error (RMSE) of position, velocity acceleration, and jerk is used as the comparison criterion in the simulation, which is defined as

(46) $$\mathrm{RMSE}\;=\;\sqrt{\frac{1}{T}\sum_{k=1}^{T}{\left(x_{k}-{\hat{x}}_{k}\right)}^{2}}$$

where $x_{k}$ denotes the real value obtained at time *k* in a certain direction, *T* represents the time of filtering iteration, and ${\hat{x}}_{k}$ is the estimated value of the filter at time *k* in a certain direction. For comparison of various aspects, the mean absolute error (MAE) is also used as the comparison criterion in the simulation, which is denoted by

(47) $$\mathrm{MAE}\;=\;\frac{1}{T}\sum_{k=1}^{T}\left|x_{k}-{\hat{x}}_{k}\right|$$

In the MSHARKF and proposed SR-SHARKF algorithms, the initial values of two constants are chosen as $c_{0}\;=\;1.2$, $c_{1}\;=\;4.5$. The parameters of the initial true state are assumed to be $X_{0}\;=[0,1,0.001,0.00005{]}^{T}$, $Y_{0}\;=[0,4,0.01,-0.0005{]}^{T}$. In addition, the square-root decomposition method (SRD), the three-segment method (TS), and the noise covariance adjustment method (NCA) are introduced to the simulation test as comparative methods to evaluate the advantages of each portion of the proposed algorithm.

**Figure 3:**
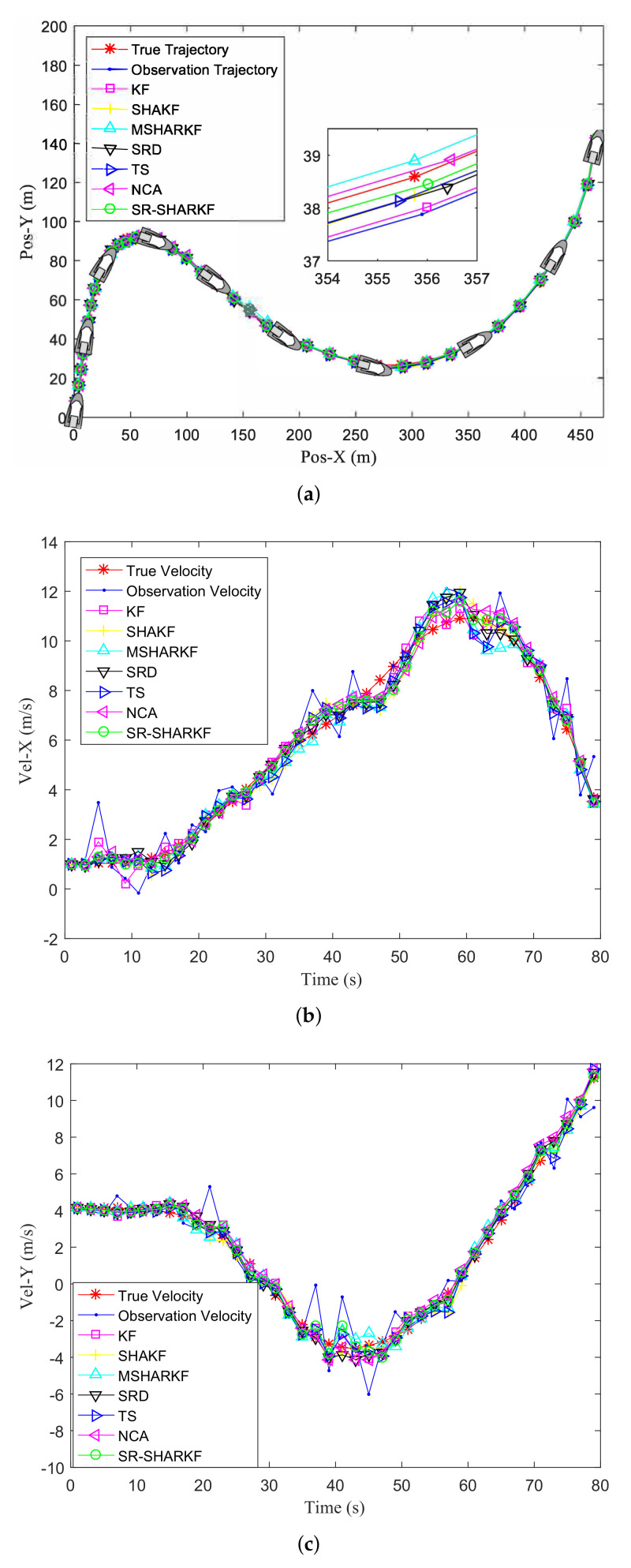
Filtering effect in terms of position and velocity. (**a**) Target trajectory. (**b**) Target velocity in the X-axis direction. (**c**) Target velocity in the Y-axis direction.

Figure 3a shows the actual trajectory, the observation trajectory of the target, and the trajectory estimated by the KF, SHAKF, MSHARKF, SRD, TS, NCA, and proposed SR-SHARKF algorithms. Moreover, the target ship is represented by the small boats in Figure 3a, and their movement on the motion trajectory will be displayed by several boats at various location points, which can reflect the real movement of the target ship more clearly. The actual ship determines the current position of the target ship at each given time by mapping the information of the target ship measured at various times to the coordinates of the Cartesian coordinate system. Figure 3b,c show the actual, observation, and filtering velocities of the X-axis and Y-axis. It can be seen from Figure 3 that the tracking effect in some states is not ideal, but in terms of the overall effect, the proposed SR-SHARKF algorithm achieves satisfactory results in filtering the measurement data compared to other algorithms. Moreover, the runtimes of the KF, SHAKF, MSHARKF, and proposed SR-SHARKF algorithms in a single step run are $9.1\times 10^{-4\;}s$, $1.16\times 10^{-3}\;s$, $2.07\times 10^{-3}\;s$, respectively.

**Figure 4:**
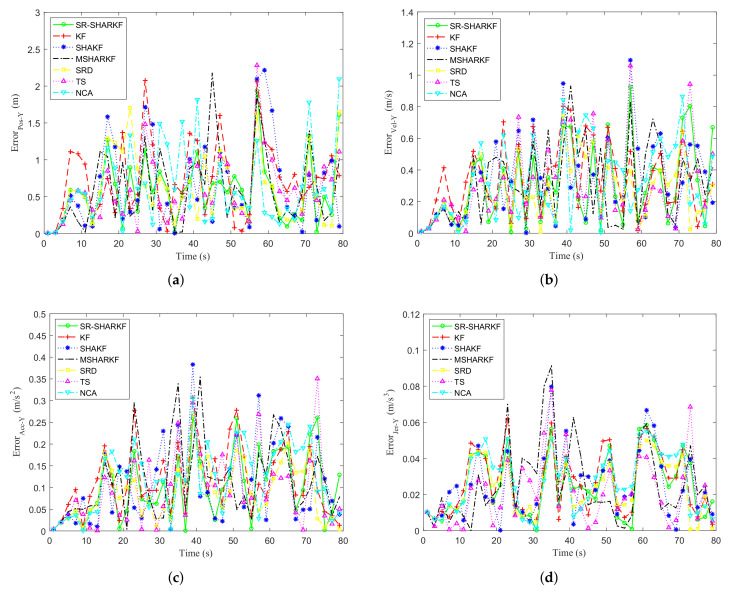
Dynamic parameter error in the X-axis direction. (**a**) Position error. (**b**) Velocity error. (**c**) Acceleration error. (**d**) Jerk error.

Taking the deviation between the true and the estimated value, the KF, SHAKF, MSHARKF, SRD, TS, NCA, and SR-SHARKF algorithms are used in radar target tracking and the simulation error curves of position, velocity, acceleration, and jerk on the X-axis are shown in Figure 4a–d. It can be seen from Figure 4 that the SRD, TS, and NCA methods for each part of the proposed algorithm have more stable error values compared to SHAKF, and the percentage of states with error values less than SHAKF is higher. As can also be seen from Figure 4, the SR-SHARKF algorithm outperforms other algorithms because the error of most states is kept at a relatively low level and is relatively stable.

According to Table 1, SRD, TS, and NCA have smaller RMSE and MAE values of position, velocity, acceleration, and jerk on the X-axis compared with SHAKF and MSHARKF, indicating that SRD, TS, and NCA can improve SHAKF better. Furthermore, the RMSE and MAE values corresponding to the proposed SR-SHARKF algorithm of position, velocity, acceleration, and jerk on the X-axis are lower compared to other algorithms, which demonstrates that it can obtain the most accurate estimation results than other algorithms in all four state variables and two aspects.

**Figure 5:**
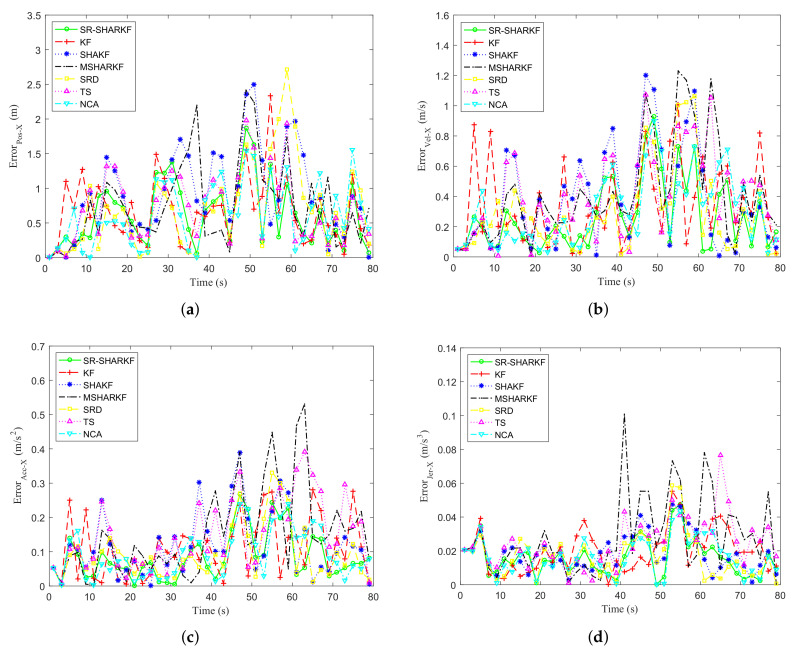
Dynamic parameter error in the Y-axis direction. (**a**) Position error. (**b**) Velocity error. (**c**) Acceleration error. (**d**) Jerk error.

In the Y-axis direction, the ship’s heading varied twice in the opposite direction. It can be seen from Figure 5 that SRD, TS, and NCA are more stable compared to other comparison algorithms, and the error of the algorithm we proposed still remains in a relatively low and stable state compared with other algorithms, which further verifies its relatively outstanding filtering effect. In addition, the RMSE and MAE values of each algorithm are recorded in Table 2, in which it can be seen that the SRD, TS, NCA, and SR-SHARKF algorithms also have obvious advantages in target tracking compared with other algorithms. These results further demonstrate the superior performance of the proposed method.

In the simulation test, compared with the previous ones, the proposed algorithm effectively reduces the appearance rate of non-positive matrices in the X-axis and Y-axis directions, which is due to the effective elimination of numerical rounding errors by the SRD method for covariance decomposition, and the interval restriction of the *Q*-matrix and *R*-matrix, as well as the transformation of non-positive to positive matrices by the NCA method together preventing the filtering divergence. Moreover, it can be seen from the simulation tests that the proposed algorithm has significantly improved the speed of adaptation of the time-varying noise covariance compared with the previous ones, which is due to the TS method being employed in the proposed algorithm to balance the observations and predicted states to improve the robustness of the algorithm.

To further verify the robustness of the proposed algorithm, another set of simulation tests was performed for the same model and different trajectories. We only compared the proposed algorithm with the KF, SHAKF, and MSHARKF algorithms. The parameters of the initial true state were chosen as $X_{0}\;=[2,1,0.001,0.0002{]}^{T}$, $Y_{0}\;=[1,2,0.0005,0.0001{]}^{T}$.

**Figure 6:**
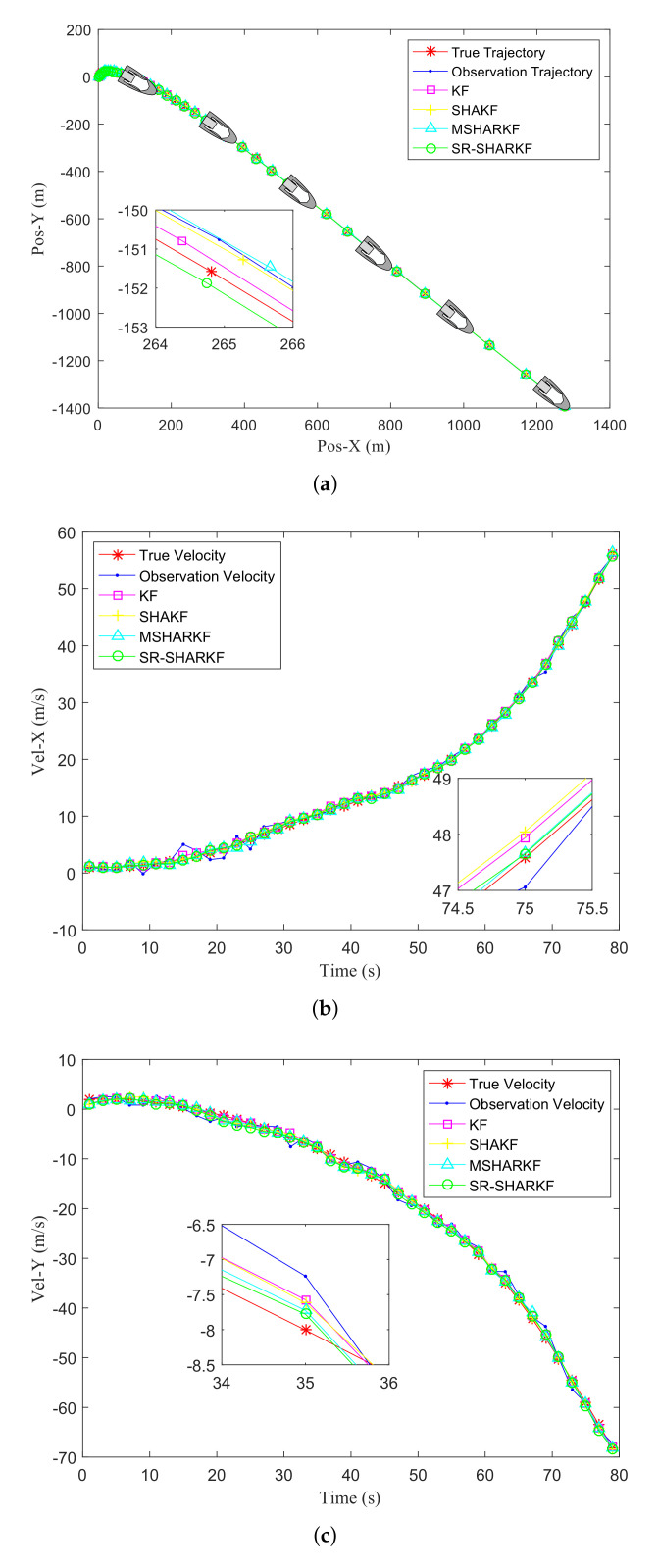
Filtering effect in terms of position and velocity. (**a**) Target trajectory. (**b**) Target velocity in the X-axis direction. (**c**) Target velocity in the Y-axis direction.

Figure 6a shows the actual trajectory, the observation trajectory of the target, and the trajectory estimated by the KF, SHAKF, MSHARKF, and proposed SR-SHARKF algorithms. Figure 6b,c show the actual, observation, and filtering velocities of the X-axis and Y-axis. From Figure 6, it can be seen that the proposed SR-SHARKF algorithm outperforms other algorithms in filtering the measurement data.

**Figure 7:**
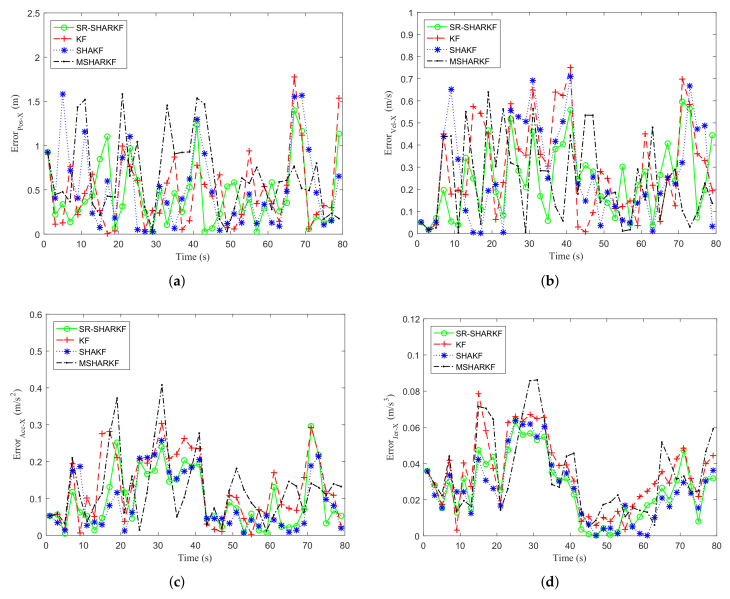
Dynamic parameter error in the X-axis direction. (**a**) Position error. (**b**) Velocity error. (**c**) Acceleration error. (**d**) Jerk error.

In the X-axis direction, the USV sails in a positive direction and has a rapid speed change. It can be observed from Figure 7 and Table 3 that the proposed SR-SHARKF has a higher percentage of smaller error values than other algorithms, and the RMSE and MAE values corresponding to the proposed SR-SHARKF algorithm of position, velocity, acceleration, and jerk are lower compared to other algorithms, which shows the superiority of the proposed algorithm over other algorithms.

**Figure 8:**
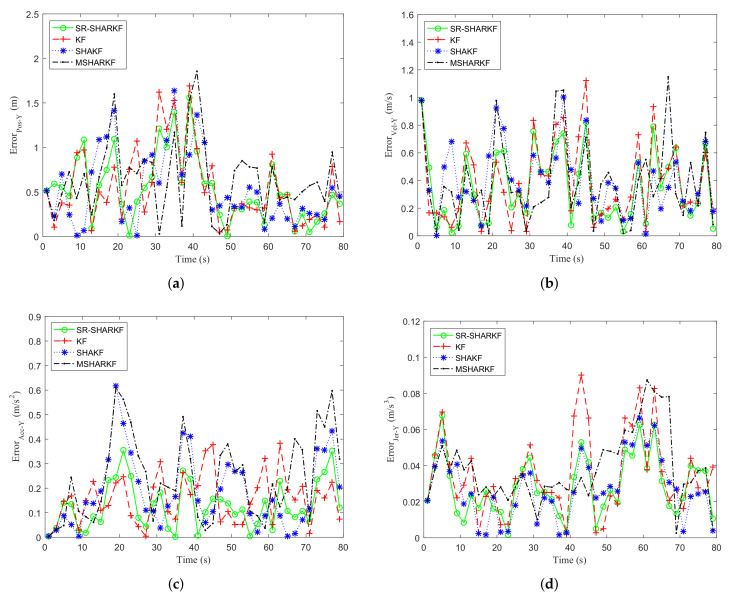
Dynamic parameter error in the Y-axis direction. (**a**) Position error. (**b**) Velocity error. (**c**) Acceleration error. (**d**) Jerk error.

In the Y-axis direction, the USV sails in a negative direction and the speed changes faster. It can be observed from Figure 8 and Table 4 that the faster the speed change, the worse the tracking effect of all algorithms. However, the proposed SR-SHARKF still outperforms other algorithms, indicating that the algorithm can also bring better performances for different trajectories.

#### 4.1.2. Simulations and Analysis of the Noise with Mixed-Gaussian Distribution

The same model as above is used to generate a maneuvering target tracking example except that the process and measurement noise are white noise satisfying the mixed-Gaussian distribution. The mixed-Gaussian process and measurement noise are given as follows:

(48) $$\left\{\begin{array}{c} q_{1k}∼0.9N\left(0,0.01\right)+0.1N\left(0,0.1\right)\\ q_{2k}∼0.9N\left(0,0.01\right)+0.1N\left(0,0.1\right)\\ q_{3k}∼0.9N\left(0,0.01\right)+0.1N\left(0,0.1\right)\\ q_{4k}∼0.9N\left(0,0.01\right)+0.1N\left(0,0.1\right)\\ r_{1k}∼0.6N\left(0,4\right)+0.4N\left(0,20\right)\\ r_{2k}∼0.6N\left(0,4\right)+0.4N\left(0,20\right) \end{array}\right.$$

In this experiment, the initial values of two constants with respect to the MSHARKF and proposed SR-SHARKF algorithms are also chosen as $c_{0}\;=\;1.2$, $c_{1}\;=\;4.5$. The parameters of the initial true state are assumed to be $X_{0}\;=[0,0,0.001,0.00001{]}^{T}$, $Y_{0}\;=[0,0,0.005,0.0005{]}^{T}$. The RMSE is employed as the comparison criterion, but the true value $x_{k}^{i}$ and the estimated value ${\hat{x}}_{k}^{i}$ at the *k*th moment of the *i*th MC run are used instead of $x_{k}$ and ${\hat{x}}_{k}$, and we replace *T* by the number of MC runs Mc. The averaged root-mean-squared error (ARMSE) of position, velocity acceleration, and jerk is also used as the evaluation criterion to test the filter performance, which is defined as

(49) $$\mathrm{ARMSE}\;=\;\sqrt{\frac{1}{TMc}\sum_{k=1}^{T}\sum_{i=1}^{Mc}{\left(x_{k}^{i}-{\hat{x}}_{k}^{i}\right)}^{2}}$$

To evaluate the dispersion of position, velocity, acceleration, and jerk for all algorithms over 1000 Monte Carlo runs, the standard deviation (STD) and the averaged STD (ASTD) as the evaluation criteria are defined as follows

(50) $$\left\{\begin{array}{c} \mathrm{STD}=\sqrt{\frac{1}{Mc}\sum_{i=1}^{Mc}\left(x_{k}^{i}-{\bar{x}}_{k}^{i}\right)}\\ \mathrm{ASTD}=\sqrt{\frac{1}{TMc}\sum_{k=1}^{T}\sum_{i=1}^{Mc}{\left(x_{k}^{i}-{\bar{x}}_{k}^{i}\right)}^{2}} \end{array}\right.$$

where ${\bar{x}}_{k}^{i}$ represents the mean of the algorithm estimates for position, velocity, acceleration, or jerk.

**Figure 9:**
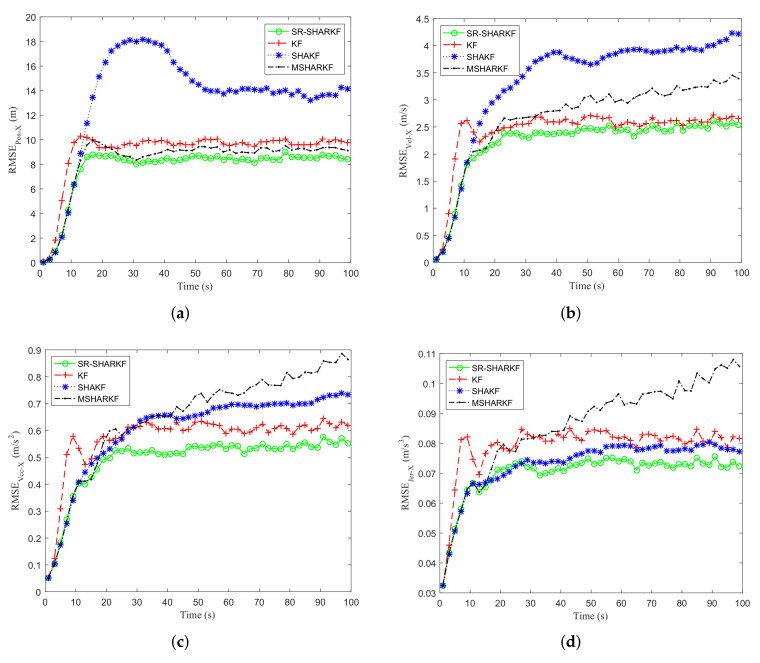
Dynamic parameter RMSE in the X-axis direction. (**a**) Position RMSE. (**b**) Velocity RMSE. (**c**) Acceleration RMSE. (**d**) Jerk RMSE.

**Figure 10:**
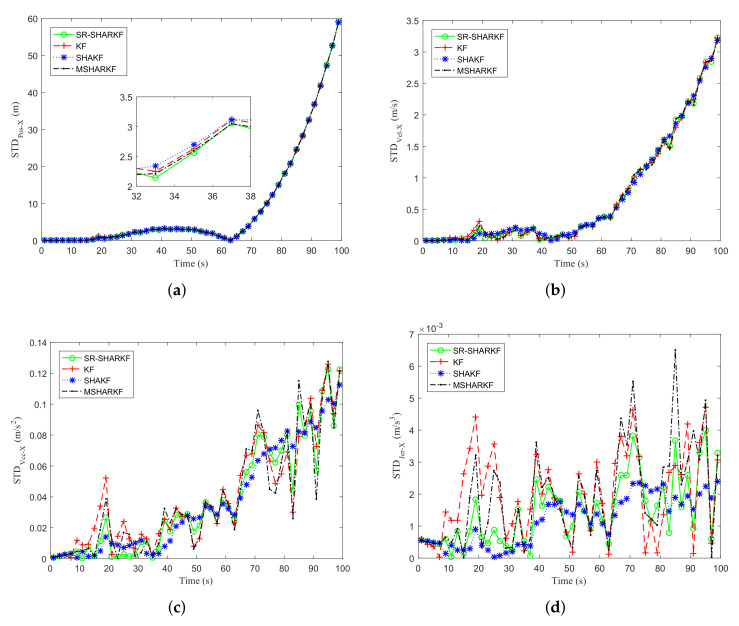
Dynamic parameter STD in the X-axis direction. (**a**) Position STD. (**b**) Velocity STD. (**c**) Acceleration STD. (**d**) Jerk STD.

Figure 9a–d and Figure 10a–d show that the RMSE and STD of position, velocity, acceleration, and jerk on the X-axis are calculated as the average and standard deviation of each state over 1000 Monte Carlo runs. It can be seen from Figure 9a–d that the RMSEs of SHAKF and MSHARKF have different degrees of divergence in terms of position, velocity, acceleration, or jerk, which is caused by their inability to estimate the noise covariance accurately in 1000 Monte Carlo simulations, while the proposed SR-SHARKF can effectively prevent the divergence and the RMSE remains relatively stable and lower, indicating that the performance of SR-SHARKF outperforms KF, SHAKF, and MSHARKF. As shown in Figure 10a–d and the ASTD of Table 5, we can see that the dispersion of the estimates of position, velocity, and acceleration for all algorithms increases with time since the changes in position, velocity, and acceleration become larger after the amount that can be changed becomes larger. The dispersions estimated by all algorithms are similar.

The ARMSE values were evaluated for all algorithms in both directions and are given in Table 5. We can see from these tables that the SR-SHARKF algorithm gives more competitive results than the KF, SHAKF, and MSHARKF algorithms.

**Figure 11:**
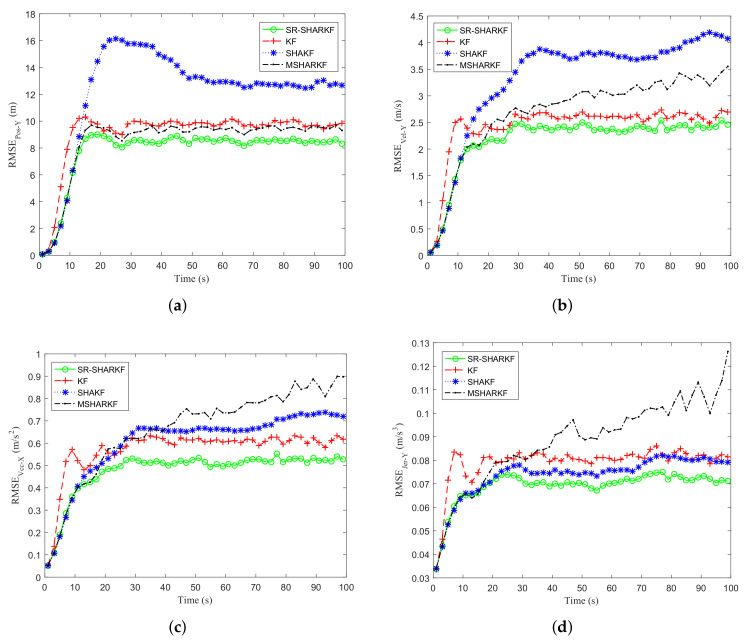
Dynamic parameter RMSE in the Y-axis direction. (**a**) Position RMSE. (**b**) Velocity RMSE. (**c**) Acceleration RMSE. (**d**) Jerk RMSE.

**Figure 12:**
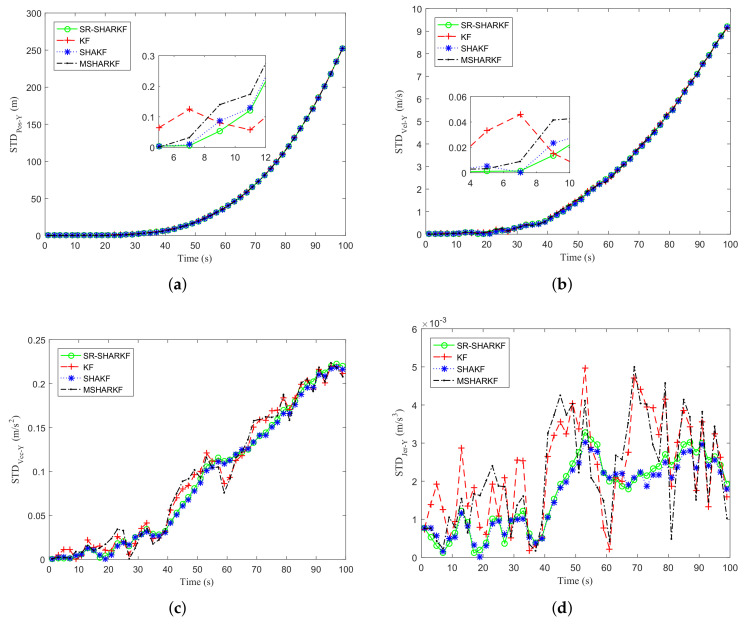
Dynamic parameter STD in the Y-axis direction. (**a**) Position STD. (**b**) Velocity STD. (**c**) Acceleration STD. (**d**) Jerk STD.

In the Y-direction, we may obtain similar results as in the X-direction by Figure 11a–d and Figure 12a–d and Table 6. It is further verified that the proposed algorithm has better performance compared to other existing algorithms.

### 4.2. Experiments and Discussion

**Figure 13:**
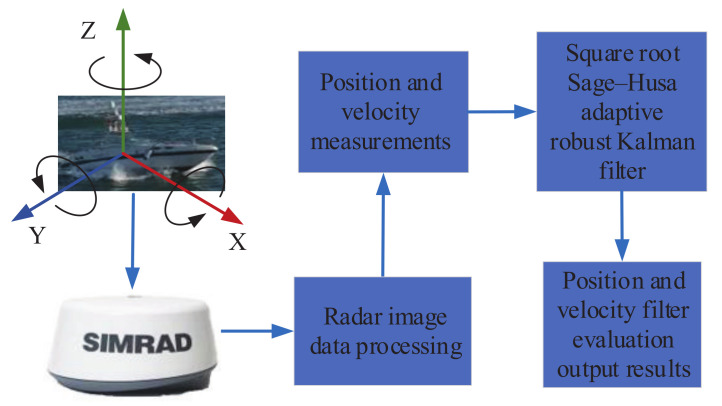
Lanxin USV experiment flow chart.

To further verify the superiority of the proposed SR-SHARKF method, the experimental data were collected by the LanXin USV in the waters along the Dalian Xinghai Cross-Sea Bridge. The experimental flow chart is shown in Figure 13. The USV’s velocity was 3.3657 m/s, the heading angle was 138.71°, and the target information sensed by the radar was within the range of 1852 m. An experimental target ship was installed with integrated navigation to save its position and velocity information as the assumed true position and velocity of the target. The assumed accuracy of position and velocity were, respectively, ±15m and ±5m/s. The SR-SHARKF algorithm proposed in this paper was compared with the tracking filtering effect of other existing algorithms.

**Figure 14:**
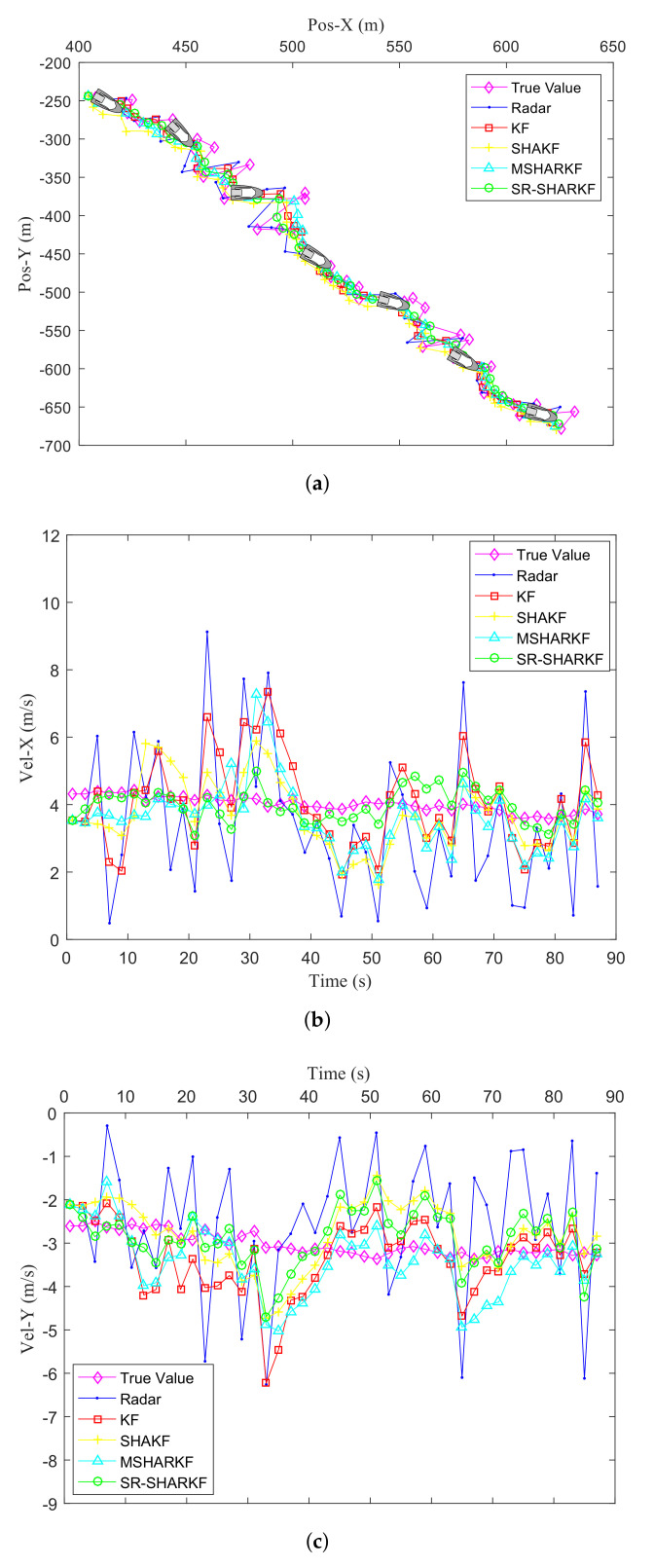
Filtering effect comparison of multiple algorithms in terms of position and velocity. (**a**) Target trajectory comparison. (**b**) Target velocity comparison in the X-axis direction. (**c**) Target velocity comparison in the Y-axis direction.

Figure 14a shows the true, radar observation trajectories of the target and the trajectory estimated by the KF, SHAKF, MSHARKF, and SR-SHARKF algorithms. It can be seen from Figure 14a that the combined filtering effect of the SR-SHARKF method in the X-axis and Y-axis directions has a better smoothing impact on the measured data, which makes it closer to the true value. Figure 14b,c show the velocity filtering effects of these algorithms in the X-axis and Y-axis directions, respectively. It can be seen from them that the SR-SHARKF algorithm can obtain a stable filtering effect on the whole. Compared with other algorithms, no matter whether in terms of trajectory or velocity, the SR-SHARKF algorithm proposed in this paper has a better filtering effect than other algorithms.

**Figure 15:**
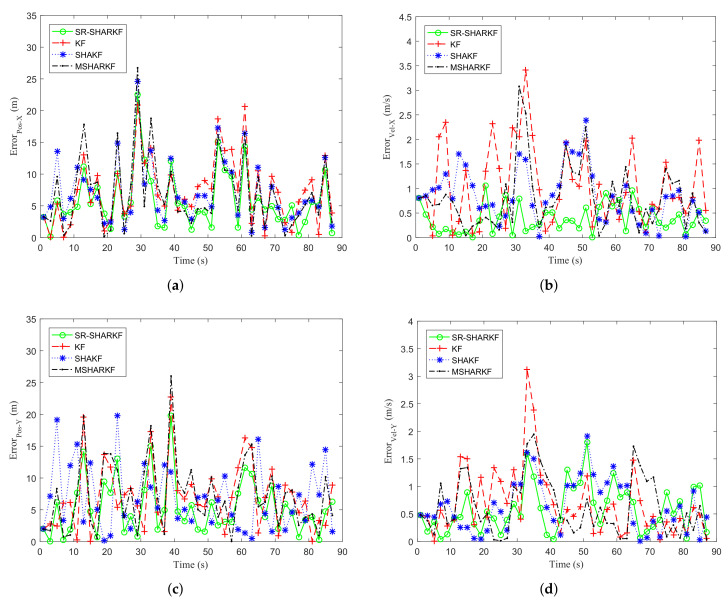
Dynamic parameter error. (**a**) Position error in the X-axis direction. (**b**) Velocity error in the X-axis direction. (**c**) Position error in the Y-axis direction. (**d**) Velocity error in the Y-axis direction.

Figure 15a–d show the error curves of position and velocity on the X-axis and the Y-axis by the KF, SHAKF, MSHARKF, and SR-SHARKF algorithms. It can be seen from Figure 15 that the majority of the states of the error curves of the SR-SHARKF algorithm are smaller than other algorithms, demonstrating that this algorithm’s tracking filtering effect is superior to other algorithms.

According to Table 7 and Table 8, the RMSE and MAE values corresponding to the proposed SR-SHARKF algorithm of position and velocity on the X-axis and the Y-axis are given. This demonstrates that the SR-SHARKF algorithm’s error for actual ship experimental data can also obtain a smaller value compared with other algorithms.

## 5. Conclusions

In this study, the SR-SHARKF algorithm was developed based on adaptive theories, which can not only solve the problem of filtering divergence triggered by numerical rounding errors, inaccurate system mathematics, and noise statistical models, but also improve the filtering accuracy. First, a novel square-root decomposition method was proposed to decompose the covariance matrix of SHAKF to ensure its non-negative definiteness. Second, an adaptive scale factor was introduced into the SHAKF algorithm and updated at each iteration. Third, the noise covariance adjustment method was applied by combining noise-biased estimation, unbiased estimation, and a fixed observation noise interval. Simulation and experimental results demonstrate that the proposed algorithm has stronger stability and robustness than the KF, SHAKF, and MSHARKF algorithms. In practical application, the proposed algorithm may be widely used in collision risk evaluation and collision avoidance, etc. In our future work, the algorithm proposed in this paper will be applied to multi-target tracking scenarios, and considering the motion of multiple targets at the same time is critical to avoiding ship collisions. 

## Figures and Tables

**Table 1:** Dynamic parameter error evaluation in the X-axis direction.

State Variables	KF		SHAKF		MSHARKF		SRD		TS		NCA		SR-SHARKF	
	RMSE	MAE	RMSE	MAE	RMSE	MAE	RMSE	MAE	RMSE	MAE	RMSE	MAE	RMSE	MAE
Pos-X	0.8274	0.6716	1.1095	0.9125	0.9762	0.7624	0.9412	0.7039	0.8836	0.7191	0.7845	0.6301	0.7806	0.6246
Vel-X	0.4448	0.3471	0.5050	0.3801	0.5251	0.4146	0.4058	0.2942	0.4956	0.4034	0.3858	0.3061	0.3543	0.2659
Vcc-X	0.1399	0.1097	0.1510	0.1184	0.1992	0.1512	0.1236	0.0951	0.1816	0.1494	0.1155	0.0922	0.1091	0.0849
Jer-X	0.0236	0.0199	0.0218	0.0186	0.0363	0.0281	0.0213	0.0169	0.0289	0.0251	0.0197	0.0164	0.0193	0.0159

**Table 2:** Dynamic parameter error evaluation in the Y-axis direction.

State Variables	KF		SHAKF		MSHARKF		SRD		TS		NCA		SR-SHARKF	
	RMSE	MAE	RMSE	MAE	RMSE	MAE	RMSE	MAE	RMSE	MAE	RMSE	MAE	RMSE	MAE
Pos-Y	0.9057	0.7444	0.8804	0.6469	0.8048	0.6150	0.7691	0.6111	0.7507	0.6038	0.8366	0.6408	0.7237	0.5737
Vel-Y	0.4121	0.3413	0.4174	0.3279	0.4079	0.3210	0.3330	0.2700	0.4089	0.3124	0.4128	0.3306	0.3964	0.3013
Vcc-Y	0.1408	0.1161	0.1433	0.1080	0.1472	0.1177	0.1119	0.0910	0.1303	0.0975	0.1449	0.1216	0.1289	0.1022
Jer-Y	0.0322	0.0266	0.0311	0.0250	0.0341	0.0259	0.0285	0.0242	0.0283	0.0215	0.0320	0.0272	0.0304	0.0250

**Table 3:** Dynamic parameter error evaluation in the X-Axis direction.

State Variables	KF		SHAKF		MSHARKF		SR-SHARKF	
	RMSE	MAE	RMSE	MAE	RMSE	MAE	RMSE	MAE
Pos-X	0.6280	0.4785	0.6847	0.5143	0.7937	0.6657	0.5786	0.4448
Vel-X	0.3694	0.2994	0.3452	0.2686	0.3066	0.2413	0.2947	0.2446
Vcc-X	0.1611	0.1318	0.1200	0.0923	0.1549	0.1256	0.1254	0.0981
Jer-X	0.0397	0.0343	0.0314	0.0257	0.0410	0.0345	0.0321	0.0270

**Table 4:** Dynamic parameter error evaluation in the Y-axis direction.

State Variables	KF		SHAKF		MSHARKF		SR-SHARKF	
	RMSE	MAE	RMSE	MAE	RMSE	MAE	RMSE	MAE
Pos-Y	0.7002	0.5509	0.6749	0.5377	0.7618	0.6432	0.6567	0.5410
Vel-Y	0.4912	0.3912	0.4785	0.4057	0.4952	0.3900	0.4438	0.3562
Vcc-Y	0.1863	0.1562	0.2360	0.1835	0.2973	0.2422	0.1597	0.1286
Jer-Y	0.0420	0.0352	0.0324	0.0272	0.0429	0.0382	0.0331	0.0287

**Table 5:** Dynamic parameter ARMSE and ASTD evaluation in the X-Axis direction.

State Variables	KF		SHAKF		MSHARKF		SR-SHARKF	
	ARMSE	ASTD	ARMSE	ASTD	ARMSE	ASTD	ARMSE	ASTD
Pos-X	9.3615	17.592	14.080	17.635	8.6543	17.638	8.0235	17.641
Vel-X	2.4925	1.1366	3.5065	1.1380	2.7888	1.1362	2.2881	1.1394
Vcc-X	0.5828	0.0526	0.6239	0.0499	0.6795	0.0525	0.5025	0.0515
Jer-X	0.0797	0.0024	0.0736	0.0014	0.0876	0.0024	0.0704	0.0018

**Table 6:** Dynamic parameter ARMSE and ASTD evaluation in the Y-axis direction.

State Variables	KF		SHAKF		MSHARKF		SR-SHARKF	
	ARMSE	ASTD	ARMSE	ASTD	ARMSE	ASTD	ARMSE	ASTD
Pos-Y	9.3762	90.525	12.807	90.260	8.8452	90.390	8.0821	90.366
Vel-Y	2.4890	3.8397	3.4720	3.8202	2.8147	3.8403	2.2455	3.8377
Vcc-Y	0.5820	0.1202	0.6239	0.1156	0.6903	0.1120	0.4878	0.1179
Jer-Y	0.0795	0.0026	0.0742	0.0018	0.0901	0.0026	0.0694	0.0019

**Table 7:** Dynamic parameter error evaluation in the X-axis direction.

State Variables	KF		SHAKF		MSHARKF		SR-SHARKF	
	RMSE	MAE	RMSE	MAE	RMSE	MAE	RMSE	MAE
Pos-X	9.1431	7.3619	8.859	7.1264	9.2173	6.9731	7.4972	5.8540
Vel-X	1.3325	1.0482	1.0261	0.8532	1.0504	0.8056	0.4804	0.3839

**Table 8:** Dynamic parameter error evaluation in the Y-axis direction.

State Variables	KF		SHAKF		MSHARKF		SR-SHARKF	
	RMSE	MAE	RMSE	MAE	RMSE	MAE	RMSE	MAE
Pos-Y	8.8864	7.0412	8.5659	6.7926	9.3207	7.3058	6.9925	5.3837
Vel-Y	0.9418	0.6898	0.8113	0.6495	0.8283	0.6283	0.7311	0.5845

## Data Availability

Not applicable.

## Abbreviations

The following abbreviations are used in this manuscript:

USV	Unmanned surface vehicle
SHAKF	Sage–Husa adaptive Kalman filter
SR-SHARKF	Square root Sage–Husa adaptive robust Kalman filter
KF	Kalman filter
MSHARKF	Modified Sage–Husa adaptive robust KF
RMSE	Root-mean-square error
MAE	Mean absolute error
SRD	Square root decomposition
TS	Three segment
ARMSE	Averaged root-mean-squared error
STD	Standard deviation
ASTD	Averaged standard deviation
